# The relationship between muscle strength and working memory in older adults: fNIRS-based evidence

**DOI:** 10.3389/fpsyg.2026.1748549

**Published:** 2026-02-17

**Authors:** Xiangbo Meng, Kaifang Liao, Zhidong Cai

**Affiliations:** 1 Department of Physical Education, Suzhou Polytechnic University, Suzhou, China; 2 Department of Sport Health, Guangdong Vocational Institute of Sport, Guangzhou, China; 3 Department of Physical Education, Suzhou University of Science and Technology, Suzhou, China

**Keywords:** 30-s sit-up, brain activation, functional near-infrared spectroscopy, grip strength, older adults, working memory

## Abstract

**Introduction:**

The association between muscle strength and cognitive function is well-established, yet its influence on working memory and related brain activation remains unclear. This study aimed to investigate the unique contributions of grip strength and 30-s sit-up to working memory performance and prefrontal cortex activation.

**Methods:**

A total of 192 older adults were recruited from nursing homes and communities. Working memory performance (reaction, time, and accuracy) was assessed using an n-back task, while hemodynamic responses were monitored in six prefrontal subregions using a 24-channel fNIRS system. Muscle strength metrics included normalized grip strength and normalized 30-s sit-up. For each dependent variable, four hierarchical regression models were constructed.

**Results:**

Normalized grip strength showed robust independent associations with high-load working memory performance (2-back reaction time: β = −0.22, *p* < 0.001; accuracy: β = 0.19, *p* = 0.002) and with activation in the dorsolateral prefrontal cortex (L-DLPFC during 2-back: β = 0.24, *p* < 0.001). When both strength metrics were included simultaneously (Model 3), normalized grip strength remained significantly associated (all *p* < 0.01), while the association of normalized 30-s sit-up became non-significant (all *p* > 0.20). The associations exhibited a clear prefrontal functional hierarchy (DLPFC > VLPFC > FPA) and cognitive load modulation pattern (0-back < 1-back < 2-back).

**Conclusion:**

The findings indicate that normalized grip strength, more so than normalized 30-s sit-up, is associated with the level of prefrontal hemodynamic activation during working memory tasks in older adults. This association is finely modulated by the functional hierarchy of the prefrontal cortex and task complexity. This highlights that standardized grip strength may be a key biomarker linked to the physical-mental health nexus, informing the development of resistance training interventions aimed at preserving cognitive health.

## Introduction

As individuals age, both their muscle strength and cognitive function decline at varying rates. Muscle mass decreases by 3–8% per decade after age 30, with the rate accelerating after age 60 (Keller and Engelhardt, 2013). Peak muscle strength at age 40 declines by an average of 16%, and by over 40.9% in individuals aged 60 and older (Volpi et al., 2004). In China, the prevalence rate of mild cognitive impairment among individuals aged 60–69, 70–79, 80–89, and 90 and older is 11.83%, 19.23%, 24.15%, and 32.46%, respectively. The prevalence rate of dementia in these age groups is 2.89%, 8.38%, 14.35%, and 31.23%, respectively (Jia et al., 2020).

Working memory, a higher-level cognitive function, is essential for the simultaneous storage and processing of information in the brain. It plays a vital role in maintaining personal functionality and independence, making it a key factor in determining the quality of life for older adults. However, working memory declines with age (Yaple et al., 2019), but it deteriorates evidently after age 60 (Elliott et al., 2011). Severe working memory impairment increases the risk of developing dementia. The prefrontal cortex is closely linked to working memory, as it is considered a critical brain region for this function. Studies have shown that the prefrontal cortex is activated during working memory tasks (Kobori et al., 2015). Additionally, the dorsolateral prefrontal cortex exhibits sustained activation during delayed response tasks, highlighting its essential role in working memory processes (Curtis and D'Esposito, 2003).

Muscle strength is a protective factor against cognitive decline, as its reduction may impair cognitive function and, consequently, affect the quality of life. A growing body of research suggests a positive correlation between muscle strength and cognitive function, indicating that maintaining muscle strength is beneficial for brain health and cognitive abilities (Marston et al., 2019). Grip strength, in particular, is considered a key indicator of brain health (Shaughnessy et al., 2020) and may share common neural substrates with higher-order cognitive functions. Older adults with higher grip strength tend to experience less age-related cognitive decline and exhibit better cognitive performance (R. P. McGrath et al., 2018). Similarly, greater quadriceps strength is associated with better cognitive performance in older adults (Chen et al., 2015).

Functional near-infrared spectroscopy (fNIRS) is a non-invasive optical technique that exploits the low absorption and high scattering properties of chromophores, such as oxygenated hemoglobin (HbO_2_), in the near-infrared spectrum (700–900 nm). Based on spectroscopy and the Beer-Lambert law, fNIRS examines the propagation of near-infrared light through brain tissue and the biochemical information conveyed by the emitted light following absorption and scattering. This enables the indirect monitoring of hemodynamic changes in the cerebral cortex, from which neural activation can be inferred via the neurovascular coupling mechanism (Expert consensus on clinical application of near-infrared brain functional imaging technology writing group, 2021). fNIRS has been widely used in cognitive function studies, as changes in cerebral blood flow can reflect cognitive performance to some extent. Experimental studies have shown that, after 16 weeks of exercise intervention, older adults exhibited shorter Stroop reaction time (RT) and reduced HbO_2_ and total hemoglobin concentrations in the left prefrontal cortex (Coetsee and Terblanche, 2017). Several studies have identified a mediating role of hemodynamics in the relationship between cardiorespiratory fitness and cognitive function in older adults (Albinet et al., 2014; Hyodo et al., 2016). As an essential component of physical fitness, muscle strength may also serve as a mediator, as explored in groundbreaking work by Herold et al. (2021). Most existing researches have focused on the relationship between muscle strength and overall cognitive function, with limited attention given to the link between muscle strength and higher-order cognitive functions, such as working memory, in older adults. Furthermore, there is a notable lack of studies exploring the relationship between muscle strength and cognitive function in older adults from a neurophysiological perspective.

This study examines the relationship between muscle strength (grip strength and the 30-s sit-up) and working memory by comparing working memory performance in participants with different levels of muscle strength. Using fNIRS, the activation characteristics of the prefrontal cortex during working memory tasks are examined to explore the influence of muscle strength on working memory from the perspective of prefrontal activation. The study hypothesizes that muscle strength is positively correlated with working memory performance, with greater muscle strength linked to better working memory performance and higher activation in related prefrontal brain regions.

##  Methods

### Participants

Participants were recruited from three nursing homes and community centers in Shanghai and Suzhou via flyers and personal introductions. Inclusion criteria:① Right-handed, regardless of gender;② Normal hearing;③ No contraindications to physical activity;④ Normal or corrected-to-normal vision. Exclusion criteria:① Severe mental illness;② Diagnosis of dementia or use of anti-cognitive impairment medications;③ Severe musculoskeletal disorders preventing independent walking;④ History of neurological disorders.

All participants were informed of the experimental procedures and provided written informed consent. Following recruitment and screening, a total of 192 older adults completed the testing. This study adhered to the ethical guidelines of the most recent version of the Declaration of Helsinki and was approved by the Ethics Committee of Suzhou University of Science and Technology (Ethics Registration Number: 240404090618.0-0001).

### Experimental procedure

**Figure 1:**
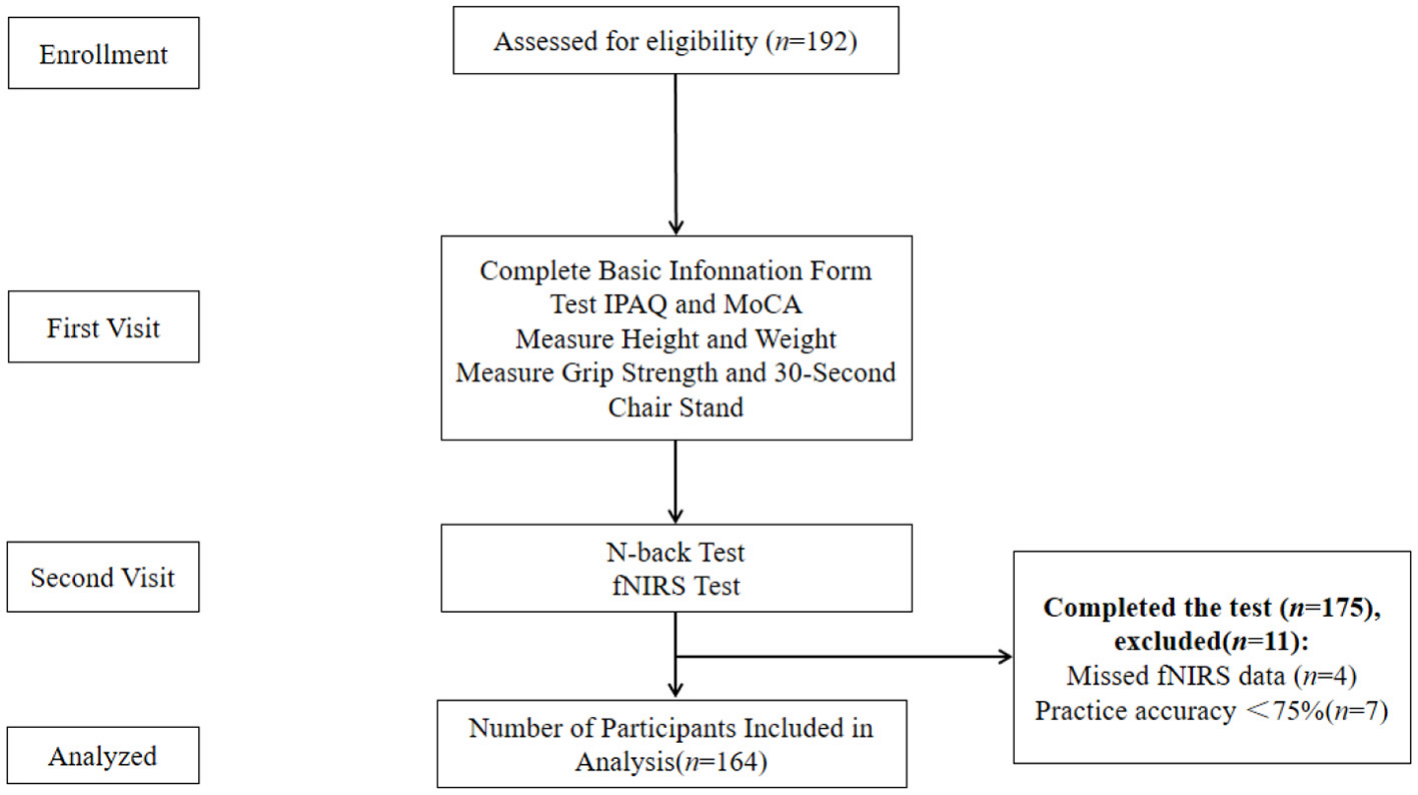
Experimental flow chart.

All participants were required to visit the laboratory twice. During the first visit, participants were briefed on the experimental procedures (Figure 1). They completed a basic information form, the Montreal Cognitive Assessment (MoCA), and the International Physical Activity Questionnaire (short form). Height, weight, grip strength, and the 30-s sit-up were measured, and participants provided informed consent. During the second visit, participants underwent the N-back test while their prefrontal cortex activation was monitored using fNIRS. The N-back task was introduced to participants, followed by a feedback-guided practice session to ensure task familiarity before the formal test. Participants were advised to avoid strenuous exercise within 24 h of each visit and refrain from consuming alcohol, coffee, or other stimulants within 2 h before testing.

### Montreal cognitive assessment

MoCA (Beijing Version) is a widely used tool for assessing participants' overall cognitive function. It includes 11 tasks across eight cognitive domains: attention, memory, executive function, language, visuospatial ability, abstract thinking, calculation, and orientation. The maximum score is 30, with higher scores indicating better overall cognitive function (Xia et al., 2021).

### Physical activity level

IPAQ (short form) is a widely recognized tool for measuring physical activity, demonstrating good reliability and validity in the Chinese population (Liang et al., 2010). This questionnaire estimates the total time and frequency of vigorous activity, moderate-intensity activity, walking, and sedentary behavior over the past 7 days. It calculates metabolic equivalents to assess physical activity across various intensities, as well as overall activity levels.

### Muscle strength

Grip strength reflects overall body strength and physical function, making it a practical and sensitive indicator (Ma et al., 2021). Grip strength was measured using an electronic handgrip dynamometer (Camry EH101, Senssun, China). Participants stood upright with their arms at their sides and wrists in a neutral position. Each hand was tested three times, with a 30-s interval between trials. The highest value from the six trials was used as the final grip strength. To account for the influence of body composition, grip strength was standardized using the following formula: normalized grip strength = grip strength (kg)/Body Mass Index (BMI) (kg/m^2^) (McGrath et al., 2020).

The 30-s sit-up test effectively evaluates lower limb muscle strength in older adults, particularly targeting muscles such as the quadriceps, semitendinosus, semimembranosus, biceps femoris, sartorius, and gastrocnemius. A preliminary model for assessing lower limb muscle strength in older adults, based on this 30-s sit-up test, has been established (Bai et al., 2020). To minimize the influence of height and weight on test outcomes, we employed a normalized 30-s sit-up for analysis (30-second sit-up/BMI).

### Working memory task

**Figure 2:**
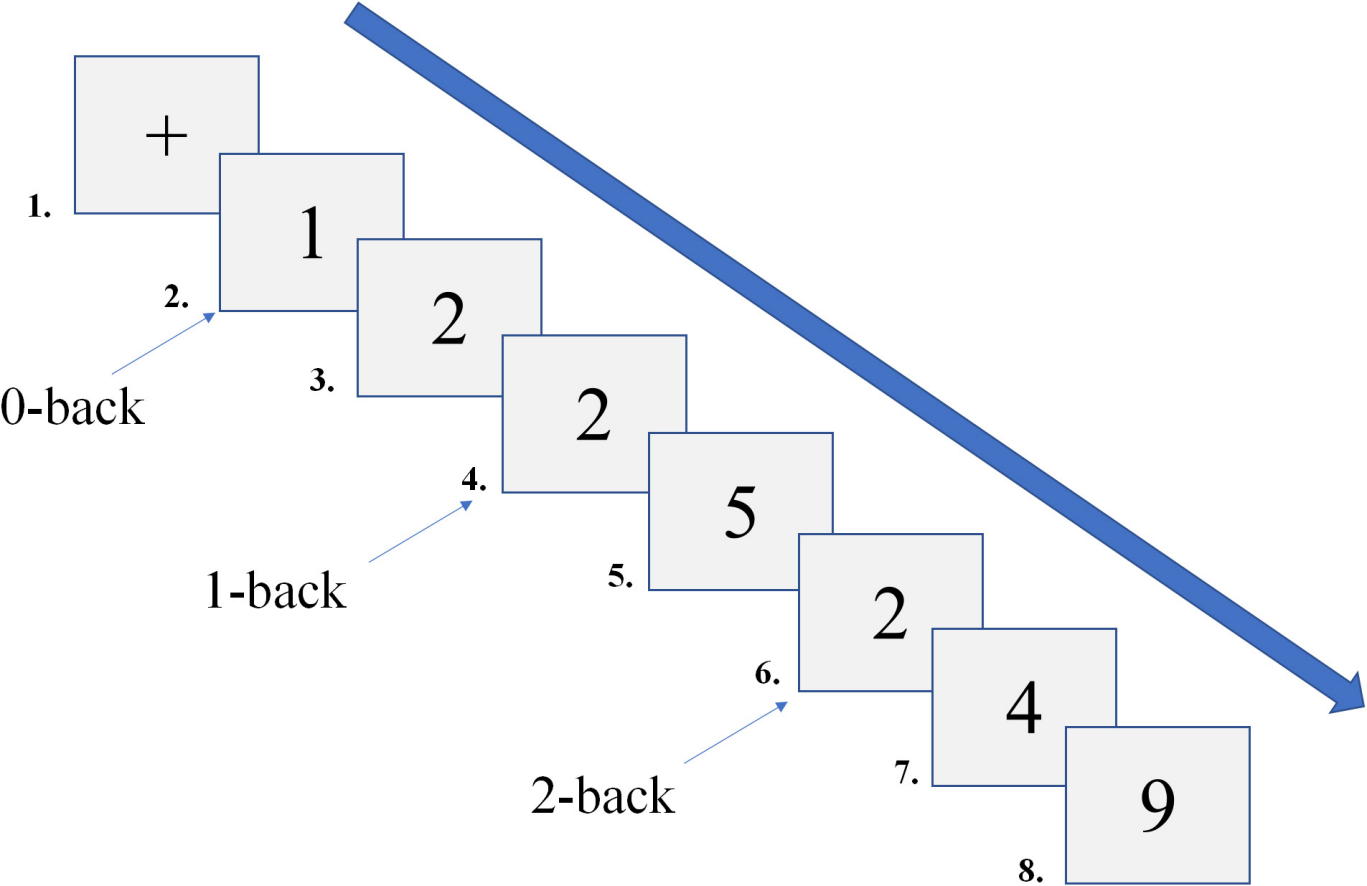
Schematic diagram of the *N*-back task.

This study employed the *N*-back paradigm to assess working memory, which was programmed using *E*-prime 2.0. The stimuli consisted of Arabic numerals, and the *N*-back task utilized a block design (Figure 2). Based on memory load, the task was divided into three conditions (0-back, 1-back, 2-back), each repeated five times. The stimuli were 3 × 3 cm numerals, randomly presented from 0 to 9 (3 target stimuli and 7 non-target stimuli). Each stimulus was presented for 500 ms, with a 2000 ms inter-stimulus interval. If no response was made within 2000 ms, the subsequent stimulus was automatically presented. In the 0-back condition, participants were instructed to compare the current number to “0.” If the numbers matched, they pressed “1”; otherwise, they pressed “2.” In the 1-back condition, starting from the second stimulus, participants judged whether the current number matched the one presented immediately prior. If they matched, they pressed “1”; otherwise, they pressed “2.” In the 2-back condition, starting from the third stimulus, participants judged whether the current number matched the one presented two steps earlier. If they matched, they pressed “1”; otherwise, they pressed “2.” During the task, participants were instructed to sit in front of a computer screen, maintaining a distance of 50 – 70 cm. Before the formal test, participants completed a practice session with feedback to familiarize themselves with the task. They were instructed to respond both quickly and accurately. Behavioral data from the *N*-back task were recorded using *E*-data, with responses faster than 200 ms (Frtusova and Phillips, 2016) or slower than 2500 ms classified as incorrect and excluded from reaction time calculations. The average RT and accuracy (ACC) for each participant were then calculated, and the cleaned data were subsequently imported into Excel.

### fNIRS measurement

**Figure 3:**
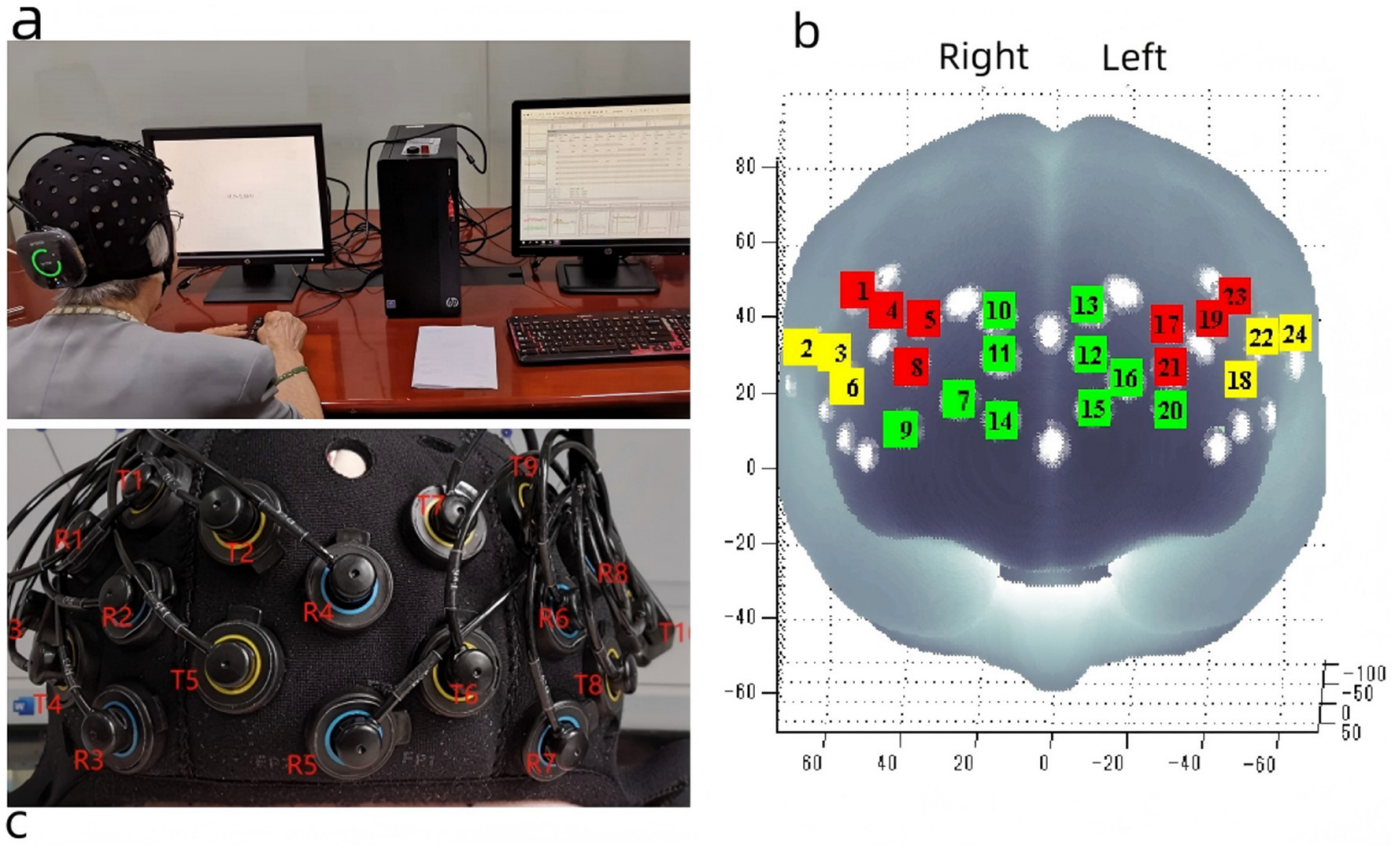
Portable near infrared brain imaging system Brite24 and ROI Setup. **(a)** Portable near infrared brain imaging system Brite24, **(b)** Region of interest, T, light emitters; R, light receptors; red square, DLPFC; yellow square, VLPFC; green square, PFA. **(c)** The distribution of light emitters and receptors. This figure is reproduced from Cai et al. (2023), under the CC BY 4.0 license.

A portable near-infrared brain imaging system, Brite24 (Artinis Medical Systems, Netherlands) (Figure 3a), was employed to measure changes in HbO_2_ concentrations during the *N*-back task. This continuous-wave near-infrared system includes 10 light sources (emitting light at wavelengths of 760 nm and 850 nm), eight detectors, and a total of 24 channels, with a source-detector separation of 30 mm. The fNIRS data were recorded and stored using Artinis' proprietary software, OxySoft 3.3.30 x 64, at a sampling frequency of 50 Hz, with the path length factor set to 6.61. The optode cap was positioned in accordance with the international 10–20 EEG placement system, with the cap's center aligned to the Cz point. In accordance with international brain region probability distribution and fNIRS optode positioning standards, the 24 channels were categorized into six regions of interest (ROI) (Figure 3b).

### fNIRS data processing

In OxySoft 3.3.30x64, the sampling frequency of the data was downsampled from 50 Hz to 10 Hz. The fNIRS data were analyzed using the NIRS-SPM (Near Infrared Spectroscopy-Statistical Parametric Mapping) toolbox (v4.1) within the Matlab R2020a environment (The MathWorks, Natick, MA, USA). Principal component analysis (PCA) was initially applied to each channel to remove physiological noise, with a parameter setting of 0.97. The Wavelet-Minimum Description Length (Wavelet-MDL) method was subsequently applied for high-pass filtering to eliminate noise (e.g., head motion, heartbeat) and baseline drift. Wavelet-MDL applies wavelet transforms to the near-infrared time series, decomposing it into various scales of bias, hemodynamic signals, and noise components. A low-pass filter was applied to the fNIRS data using the hemodynamic response function. A general linear model was constructed to model task effects by integrating task-related reference waveforms and inferring parameter estimates. Temporal autocorrelation was corrected using the precoloring method during this analysis (Bai et al., 2016).

### Statistical analysis

Statistical analysis was conducted using IBM SPSS Statistics 22.0 (IBM, Chicago, USA). Continuous variables that did not conform to a normal distribution were expressed as medians with interquartile ranges, while categorical variables were presented as percentages. Four hierarchical regression models were constructed for each dependent variable (behavioral and neural activation measures). Model 0 (Baseline Model): Included only control variables: age, sex, and Montreal Cognitive Assessment (MoCA) score. Model 1: Baseline model + normalized grip strength. Model 2: Baseline model + normalized 30-s sit-up. Model 3: Baseline model + normalized grip strength + normalized 30-s sit-up. All statistical tests were two-tailed, and statistical significance was defined as *P* < 0.05. Multiple comparisons of the behavioral data and fNIRS data were corrected using the False Discovery Rate (FDR) method, with the significance level set at *q* < 0.05.

## Results

### Participant characteristics

A total of 192 older adults were recruited, with 175 completing all tests. Ultimately, 164 participants were included in the final analysis, consisting of 103 females (62.8%) and 61 males (37.2%), with a mean age of 81.3. The mean MoCA score was 23.5 (See Table 1 for details).

**Table 1:** Characteristics of the study participants (mean ± standard deviation).

**Characteristic**	**Total (*N* = 164)**	**Female (*n* = 103)**	**Male (*n* = 61)**
Age, years	81.3 ± 1.1	81.8 ± 1.0	80.5 ± 1.2
Gender, females %	100%	62.8%	37.2%
BMI, kg/m^2^	22.9 ± 0.5	23.1 ± 0.5	22.6 ± 0.5
MoCA, score	23.5 ± 0.8	22.9 ± 0.8	24.3 ± 0.9
IPAQ, MET-min/week	1322 ± 151	1301 ± 142	1358 ± 163
Education level, years	8.7 ± 3.6	8.5 ± 3.9	8.9 ± 3.8
Grip strength, kg	24.1 ± 5.8	20.3 ± 4.1	30.5 ± 5.2
Normalized grip strength, kg/BMI	1.05 ± 0.25	0.88 ± 0.18	1.35 ± 0.22
30-sec sit-up, counts	12.4 ± 4.3	10.8 ± 3.9	15.2 ± 4.5
Normalized 30-sec sit-up, counts/BMI	0.54 ± 0.19	0.47 ± 0.17	0.67 ± 0.20
RT0 (ms)	652 ± 85	665 ± 88	631 ± 78
RT1 (ms)	787 ± 102	758 ± 105	734 ± 94
RT2 (ms)	823 ± 125	845 ± 128	788 ± 118
ACC0 (%)	98.5 ± 2.1	98.2 ± 2.3	99.0 ± 1.8
ACC1 (%)	85.8 ± 4.5	84.9 ± 4.8	87.3 ± 3.9
ACC2 (%)	77.3 ± 7.2	78.7 ± 7.5	73.0 ± 6.8

### The association among muscle strength, working memory performance, and prefrontal cortex activation

To examine the unique and combined contribution of grip strength and 30-s sit-up to working memory performance and its neural underpinnings, we conducted a series of hierarchical regression analyses.Models 1–3 were adjusted for age, sex, MoCA score, IPAQ and years of education. Model 0 included covariates only. Model 1 added normalized grip strength to Model 0, and Model 2 added normalized 30-s sit-up. Model 3 included both strength measures simultaneously to evaluate their independent predictive power.

### Reaction time metrics

For reaction times (Table 2) in the 0-back, 1-back, and 2-back tasks, baseline models explained 19%, 20%, and 21% of variance (adjusted *R2*), respectively. Adding normalized grip strength (Model 1) significantly improved model fit (Δ*R2* = 0.04, 0.07, and 0.08, respectively; all *p* < 0.001), with significant negative predictive effects for normalized grip strength (standardized coefficients β = −0.20, −0.26, and −0.28, respectively; all *p* ≤ 0.002). By contrast, the incremental contribution of normalized 30-s sit-ups (Model 2) was smaller (Δ*R2* = 0.02, 0.04, and 0.04, respectively; *p* ≤ 0.014), with weaker predictive effects (β = −0.14, −0.20, and −0.19, respectively).

**Table 2:** Hierarchical regression results for reaction time prediction.

**Task**	**Model**	**Adjusted *R^2^***	**Δ*R^2^* (vs. model 0)**	***F*(*p*)**	**NGS β [95%CI] (*p*)**	**30NSUP β [95%CI] (*p*)**
RT0	0	0.19	—	—	—	—
	1	0.23	**0.04 (*****p*** **=** **0.002)**	*F* (1,96) = 10.24 (*p* = 0.002)	−0.20 [−0.31–0.09] (*p* =0.002)	—
	2	0.21	0.02 (*p* = 0.045)	*F* (1,96) = 4.06 (*p* = 0.045)	—	−0.14 [−0.25 −0.03] (*p* = 0.045)
	3	0.24	0.05 (*p* = 0.002)	*F* (2, 95) = 5.89 (*p* = 0.004)	−0.18 [−0.29–0.07] (*p* = 0.003)	−0.05 [−0.16, 0.06] (*p* = 0.45)
RT1	0	0.20	—	—	—	—
	1	0.27	0.07 (*p* < 0.001)	*F* (1.96) = 16.00 (*p* < 0.001)	−0.26 [−0.37–0.15] (*p* < 0.001)	—
	2	0.24	0.04 (*p* = 0.009)	*F* (1.96) = 7.29 (*p* = 0.009)	—	−0.20 [−0.31 −0.09] (*p* = 0.009)
	3	0.28	0.08 (*p* < 0.001)	*F* (2.95) = 9.78 (*p* < 0.001)	−0.23 [–0.34 –0.12] (*p* < 0.001)	−0.06 [−0.17, 0.05] (*p* = 0.38)
RT2	0	0.21	—	—	—	—
	1	0.29	0.08 (*p* < 0.001)	*F* (1.96) = 18.29 (*p* < 0.001)	−0.28 [−0.39 −0.17] (*p* < 0.001)	—
	2	0.25	0.04 (*p* = 0.014)	*F* (1.96) = 6.25 (*p* = 0.014)	—	−0.19 [−0.30 −0.08] (*p* = 0.014)
	3	0.30	0.09 (*p* < 0.001)	*F* (2.95) = 10.67 (*p* < 0.001)	−0.22 [−0.33, −0.11] (*p* < 0.001)	−0.08 [−0.19, 0.03] (*p* = 0.20)

*RT0, RT1, RT2* represents the reaction time for the 0-back, 1-back, 2-back, respectively. NGS= normalized grip strength, 30NSUP= normalized 30-sec sit-up

The key finding was in Model 3: when both normalized grip strength and normalized 30-s sit-up were included simultaneously, normalized grip strength retained robust and significant predictive effects (β = −0.18, −0.23, −0.22, respectively; all *p* ≤ 0.003), while the contribution of normalized 30-s sit-up became nonsignificant (all *p* ≥0.20). The variance inflation factors were all below 1.5, indicating no multicollinearity. This pattern suggests that the physiological characteristics captured by normalized grip strength substantially overlapped with the association between normalized 30-s sit-up and reaction time performance.

### Accuracy metrics

For accuracy (Table 3), normalized grip strength showed a significant unique predictive effect for the high load tasks (1-back and 2-back) (Model 1: ΔR^2^ = 0.04 and 0.05, respectively, *p* ≤ 0.003; β = 0.20 and 0.22, respectively). In Model 3, the predictive effects of normalized grip strength on ACC1, and ACC2 remained significant (β = 0.16 and 0.19, respectively, *p* ≤ 0.012), while the effect of normalized 30-s sit-up was not significant. For 0-back accuracy, the predictive effects were not statistically significant in all models (all *p* ≥ 0.05).

**Table 3:** Hierarchical regression results for accuracy prediction.

**Task**	**Model**	**Adjusted *R^2^***	**Δ*R^2^* (vs.model0)**	***F* (*p*-value)**	**NGS β [95%CI] (*p*)**	**30NSUP β [95%CI] (*p*)**
ACC0	0	0.14	—	—	—	—
	1	0.16	0.02 (*p* = 0.058)	*F* (1.96) = 3.67 (*p* = 0.058)	0.14 [−0.01, 0.29] (*p* = 0.058)	—
	2	0.15	0.01 (*p* = 0.18)	*F* (1.96) = 1.80 (*p* = 0.18)	—	0.10 [−0.05, 0.25] (*p* = 0.18)
	3	0.17	0.03 (*p* = 0.11)	*F* (2.95) = 2.11 (*p* = 0.13)	0.12 [−0.03, 0.27] (*p* = 0.12)	0.02 [−0.13, 0.17] (*p* = 0.80)
ACC1	0	0.16	—	—	—	—
	1	0.20	0.04 (p = 0.008)	*F* (1.96) = 7.29 (*p* = 0.008)	0.20 [0.05, 0.35] (*p* = 0.008)	—
	2	0.18	0.02 (*p* = 0.065)	*F* (1.96) = 3.46 (*p* = 0.065)	—	0.14 [−0.01, 0.29] (*p* = 0.065)
	3	0.21	0.05 (*p* = 0.009)	*F* (2.95) = 4.01 (*p* = 0.022)	0.16 [0.01, 0.31] (*p* = 0.012)	0.04 [−0.11, 0.19] (*p* =0.60)
ACC2	0	0.17	—	—	—	—
	1	0.22	0.05 (*p* = 0.003)	*F* (1.96) = 9.00 (*p* = 0.003)	0.22 [0.07, 0.37] (*p* = 0.003)	—
	2	0.19	0.02 (*p* = 0.045)	*F* (1.96) = 4.06 (*p* = 0.045)	—	0.14 [−0.01, 0.29] (*p* = 0.045)
	3	0.23	0.06 (*p* = 0.003)	*F* (2.95) = 5.89 (*p* = 0.004)	0.19 [0.04, 0.34] (*p* = 0.004)	0.05 [−0.10, 0.20] (*p* = 0.52)

ACC0, ACC1, and ACC2 represent accuracy rates (percentage correct) for 0-back, 1-back, and 2-back, respectively.

### Associations between muscle strength and prefrontal cortex neural activation

We further examined the associations between muscle strength and neural activation in each of the prefrontal sub-regions under different cognitive loads. The analysis across six brain regions (left/right dorsolateral prefrontal cortex, left/right ventrolateral prefrontal cortex, and left/right frontopolar area) revealed a consistent pattern.

#### Dorsolateral prefrontal cortex

For the L-DLPFC, normalized grip strength showed a significant unique association with activation in the 0-back, 1-back, and 2-back tasks (Model 1: Δ*R*^2^ = 0.05, 0.10, 0.12, respectively; all *p* ≤ 0.008; β = 0.15, 0.22, 0.28, respectively). In Model 3, the association of normalized grip strength remained robust (β = 0.15, 0.22, 0.24, respectively; all *p* ≤ 0.009), while the association of normalized 30-s sit-ups was nonsignificant. The R-DLPFC showed a similar but slightly weaker pattern. (See Tables 4, 5 for details)

**Table 4:** Hierarchical regression results for left dorsolateral prefrontal cortex activation.

**Task**	**Model**	**Adjusted *R^2^***	**Δ*R^2^* (vs. Model 0)**	***F* (*p*-value)**	**NGS β [95%CI] (*p*)**	**30NSUP β [95%CI] (*p*)**
0-back	0	0.08	—	—	—	—
	1	0.13	0.05 (*p* = 0.008)	*F* (1.96) = 7.29 (*p* = 0.008)	0.15 [0.04, 0.26] (*p* = 0.008)	—
	2	0.10	0.02 (*p* = 0.10)	*F* (1.96) = 2.71 (*p* = 0.10)	—	0.11 [0.00, 0.22] (*p* = 0.10)
	3	0.13	0.05 (*p* = 0.015)	*F* (295) = 4.41 (*p* = 0.015)	0.15 [0.04, 0.26] (*p* = 0.009)	0.03 [−0.08, 0.14] (*p* = 0.70)
1-back	0	0.12	—	—	—	—
	1	0.22	0.10 (*p* < 0.001)	*F* (1.96) = 16.00 (*p* < 0.001)	0.22 [0.11, 0.33] (*p* < 0.001)	—
	2	0.17	0.05 (*p* = 0.009)	*F* (1.96) = 7.29 (*p* = 0.009)	—	0.15 [0.04, 0.26] (*p* = 0.009)
	3	0.22	0.10 (*p* < 0.001)	*F* (2.95) = 9.78 (*p* < 0.001)	0.22 [0.11, 0.33] (*p* < 0.001)	0.05 [−0.06, 0.16] (*p* = 0.50)
2-back	0	0.10	—	—	—	—
	1	0.22	0.12 (*p* < 0.001)	*F* (1.96) = 18.29 (*p* < 0.001)	0.28 [0.17, 0.39] (*p* < 0.001)	—
	2	0.15	0.05 (*p* = 0.014)	*F* (1.96) = 6.25 (*p* = 0.014)	—	0.17 [0.06, 0.28] (*p* = 0.014)
	3	0.22	0.12 (*p* < 0.001)	*F* (2.95) = 9.15 (*p* < 0.001)	0.24 [0.12, 0.36] (*p* < 0.001)	0.05 [−0.07, 0.17] (*p* = 0.55)

**Table 5:** Hierarchical regression results for right dorsolateral prefrontal cortex activation

**Task**	**Model**	**Adjusted R^2^**	**Δ*R^2^* (vs. Model 0)**	***F* (*p*-value)**	**NGS β [95%CI] (*p*)**	**30NSUP β [95%CI] (*p*)**
0-back	0	0.06	—	—	—	—
	1	0.10	0.04 (*p* = 0.028)	*F* (1.96) = 4.92 (*p* = 0.028)	0.12 [0.01, 0.23] (*p* = 0.028)	—
	2	0.07	0.01 (*p* = 0.20)	*F* (1.96) = 1.66 (*p* = 0.20)	—	0.09 [−0.02, 0.20] (*p* = 0.20)
	3	0.10	0.04 (*p* = 0.045)	*F* (2.95) = 3.16 (*p* = 0.047)	0.11 [0.00, 0.22] (*p* = 0.048)	0.02 [−0.09, 0.13] (*p* = 0.80)
1-back	0	0.10	—	—	—	—
	1	0.19	0.09 (*p* < 0.001)	*F* (1.96) = 12.25 (*p* < 0.001)	0.19 [0.08, 0.30] (*p* < 0.001)	—
	2	0.14	0.04 (*p* = 0.025)	*F* (1.96) = 5.06 (*p* = 0.025)	—	0.12 [0.01, 0.23] (*p* = 0.025)
	3	0.19	0.09 (*p* < 0.001)	*F* (2.95) = 7.11 (*p* = 0.001)	0.19 [0.08, 0.30] (*p* < 0.001)	0.04[−0.07, 0.15] (*p* = 0.60)
2-back	0	0.09	—	—	—	—
	1	0.21	0.12 (*p* < 0.001)	*F* (1.96) = 18.29 (*p* < 0.001)	0.25 [0.14, 0.36] (*p* < 0.001)	—
	2	0.15	0.06 (*p* = 0.006)	*F* (1.96) = 7.84 (*p* = 0.006)	—	0.15 [0.04, 0.26] (*p* =0.006)
	3	0.21	0.12 (*p* < 0.001)	*F* (2.95) = 9.78 (*p* < 0.001)	0.23 [0.12, 0.34] (*p* < 0.001)	0.06 [−0.05, 0.17] (*p* = 0.45)

**Ventrolateral Prefrontal Cortex**

The left ventrolateral prefrontal cortex (L-VLPFC) was not significantly associated with muscle strength in the 0-back. In contrast, normalized grip strength showed a significant association in the 1- and 2-back tasks (Model 1: ΔR^2^ = 0.06 and 0.10, respectively, *p* ≤ 0.003; β = 0.16 and 0.21, respectively).

Model 3 again confirmed that normalized grip strength was the dominant factor. The pattern of results for the right ventrolateral prefrontal cortex (R-VLPFC) was similar but with smaller effect sizes. (See Tables 6, 7 for details)

**Table 6:** Hierarchical regression results for right ventrolateral prefrontal cortex activation

**Task**	**Model**	**Adjusted *R^2^***	**Δ *R^2^* (vs. Model 0)**	***F* (*p*-value)**	**NGS β[95%CI] (*p*)**	**30NSUP β[95%CI] (*p*)**
0-back	0	0.04	—	—	—	—
	1	0.05	0.01 (*p* = 0.35)	*F* (1.96) = 0.88 (*p* = 0.35)	0.06 [−0.05, 0.17] (*p* =0.35)	—
	2	0.04	0.00 (*p* = 0.65)	*F* (1.96) = 0.21 (*p* = 0.65)	—	0.03 [−0.08, 0.14] (*p* = 0.65)
	3	0.05	0.01 (*p* = 0.55)	*F* (2.95) = 0.60 (*p* = 0.55)	0.06 [−0.05, 0.17] (*p* = 0.35)	0.01 [-0.10, 0.12] (*p* = 0.90)
1-back	0	0.07	—	—	—	—
	1	0.12	0.05 (*p* = 0.010)	*F* (1.96) = 6.76 (*p* = 0.010)	0.13 [0.02, 0.24] (*p* = 0.010)	—
	2	0.09	0.02 (*p* = 0.085)	*F* (1.96) = 3.06 (*p* = 0.085)	—	0.11 [0.00, 0.22] (*p* = 0.085)
	3	0.12	0.05 (*p* = 0.020)	*F* (2.95) = 4.01 (*p* = 0.022)	0.13 [0.02, 0.24] (*p* = 0.012)	0.03 [−0.08, 0.14] (*p* = 0.70)
2-back	0	0.06	—	—	—	—
	1	0.15	0.09 (*p* < 0.001)	*F* (1.96) = 12.25 (*p* < 0.001)	0.18 [0.07, 0.29] (*p* < 0.001)	—
	2	0.10	0.04 (*p* = 0.025)	*F* (1.96) = 5.06 (*p* = 0.025)	—	0.12 [0.01, 0.23] (*p* = 0.025)
	3	0.15	0.09 (*p* < 0.001)	*F* (2.95) = 6.53 (*p* = 0.002)	0.18 [0.07, 0.29] (*p* < 0.001)	0.04 [−0.07, 0.15] (*p* = 0.60)

**Table 7:** Hierarchical regression results for left ventrolateral prefrontal cortex activation.

**Task**	**Model**	**Adjusted R^2^**	**ΔR^2^ (vs. Model 0)**	***F* (*p*-value)**	**NGS β[95%CI] (*p*)**	**30NSUP β[95%CI] (*p*)**
0-back	0	0.05	—	—	—	—
	1	0.07	0.02 (*p* = 0.12)	*F* (1.96) = 2.46 (*p* = 0.12)	0.10 [−0.01, 0.21] (*p* = 0.12)	—
	2	0.06	0.01 (*p* = 0.25)	*F* (1.96) = 1.33 (*p* = 0.25)	—	0.08 [−0.03, 0.19] (*p* = 0.25)
	3	0.07	0.02 (*p* = 0.25)	*F* (2.95) = 1.64 (*p* = 0.20)	0.09 [−0.02, 0.20] (*p* = 0.12)	0.01 [−0.10, 0.12](*p* = 0.90)
1-back	0	0.08	—	—	—	—
	1	0.14	0.06 (*p* = 0.003)	*F* (1.96) = 9.00 (*p* = 0.003)	0.16 [0.05, 0.27] (*p* = 0.003)	—
	2	0.11	0.03 (*p* = 0.045)	*F* (1.96) = 4.06 (*p* = 0.045)	—	0.11 [0.00, 0.22] (*p* = 0.045)
	3	0.14	0.06 (*p* = 0.006)	*F* (295) = 4.84 (*p* = 0.010)	0.15 [0.04, 0.26] (*p* = 0.005)	0.04 [−0.07, 0.15] (*p* = 0.60)
2-back	0	0.07	—	—	—	—
	1	0.17	0.10 (*p* < 0.001)	*F* (1.96) = 14.44 (*p* < 0.001)	0.21 [0.10, 0.32] (*p* < 0.001)	—
	2	0.12	0.05 (*p* = 0.014)	*F* (1.96) = 6.25 (*p* = 0.014)	—	0.13 [0.02, 0.24] (*p* = 0.014)
	3	0.17	0.10 (*p* < 0.001)	*F* (2.95) = 7.84 (*p* = 0.001)	0.20 [0.09, 0.31] (*p* < 0.001)	0.05 [−0.06, 0.16] (*p* = 0.50)

**Frontopolar Area**

The frontopolar area (FPA) showed the weakest associations with muscle strength (Tables 8, 9). Neither left nor right FPA showed significant associations in the 0-back task. In the 1-back and 2-back tasks, normalized grip strength showed a small but significant association (e.g., L-FPA in 2-back: Model 1 Δ*R*^2^ = 0.07, β = 0.17, *p* = 0.002), while normalized 30-sec sit-ups were non-significant across all models in analysis 3.

**Table 8:** Hierarchical regression results for left frontopolar area activation

**Task**	**Model**	**Adjusted *R^2^***	**Δ*R^2^* (vs. Model 0)**	***F* (*p*-value)**	**NGS β [95%CI] (*p*)**	**30NSUP β [95%CI] (*p*)**
0-back	0	0.03	—	—	—	—
	1	0.04	0.01 (*p* = 0.40)	*F* (1.96) = 0.72 (*p* = 0.40)	0.07 [−0.04, 0.18] (*p* = 0.40)	—
	2	0.03	0.00 (*p* = 0.65)	*F* (1.96) = 0.21 (*p* = 0.65)	—	0.03 [−0.08, 0.14] (*p* = 0.65)
	3	0.04	0.01 (*p* = 0.55)	*F* (2.95) = 0.60 (*p* = 0.55)	0.05 [−0.06, 0.16] (*p* = 0.40)	0.02 [−0.09, 0.13] (*p* = 0.80)
1-back	0	0.05	—	—	—	—
	1	0.09	0.04 (*p* = 0.025)	*F* (1.96) = 5.06 (*p* = 0.025)	0.12 [0.01, 0.23] (*p* = 0.025)	—
	2	0.07	0.02 (*p* = 0.10)	*F* (1.96) = 2.71 (*p* = 0.10)	—	0.11 [0.00, 0.22] (*p* = 0.10)
	3	0.09	0.04 (*p* = 0.045)	*F* (2.95) = 3.16 (*p* = 0.047)	0.12 [0.01, 0.23] (*p* = 0.028)	0.03 [−0.08, 0.14] (*p* = 0.70)
2-back	0	0.04	—	—	—	—
	1	0.11	0.07 (*p* = 0.002)	*F* (1.96) = 10.24 (*p* = 0.002)	0.17 [0.06, 0.28] (*p* = 0.002)	—
	2	0.08	0.04 (*p* = 0.028)	*F* (1.96) = 4.92 (*p* = 0.028)	—	0.12 [0.01, 0.23] (*p* = 0.028)
	3	0.11	0.07 (*p* = 0.003)	*F* (2.95) = 5.40 (*p* = 0.006)	0.16 [0.05, 0.27] (*p* = 0.003)	0.04 [−0.07, 0.15] (*p* = 0.60)

**Table 9:** Hierarchical regression results for right frontopolar area activation.

**Task**	**Model**	**Adjusted *R^2^***	**Δ*R^2^* (vs. Model 0)**	***F* (*p*-value)**	**NGS β [95%CI] (*p*)**	**30NSUP β [95%CI] (*p*)**
0-back	0	0.02	—	—	—	—
	1	0.03	0.01 (*p* = 0.50)	*F* (1.96) = 0.46 (*p* = 0.50)	0.06 [−0.05, 0.17] (*p* = 0.50)	—
	2	0.02	0.00 (*p* = 0.75)	*F* (1.96) = 0.10 (*p* = 0.75)	—	0.02 [−0.09, 0.13] (*p* = 0.75)
	3	0.03	0.01 (*p* = 0.65)	*F* (2.95) = 0.42 (*p* = 0.66)	0.04 [-0.07, 0.15] (*p* =0.50)	0.02 [−0.09, 0.13] (*p* = 0.80)
1-back	0	0.04	—	—	—	—
	1	0.08	0.04 (*p* = 0.018)	*F* (1.96) = 5.76 (*p* = 0.018)	0.11 [0.00, 0.22] (*p* = 0.018)	—
	2	0.06	0.02 (*p* = 0.12)	*F* (1.96) = 2.46 (*p* = 0.12)	—	0.10 [−0.01, 0.21] (*p* = 0.12)
	3	0.08	0.04 (*p* = 0.030)	*F* (2.95) = 3.55 (*p* = 0.033)	0.11 [0.00, 0.22] (*p* = 0.020)	0.03 [−0.08, 0.14] (*p* = 0.70)
2-back	0	0.03	—	—	—	—
	1	0.10	0.07 (*p* = 0.001)	*F* (1.96) = 12.25 (*p* = 0.001)	0.15 [0.04, 0.26] (*p* = 0.001)	—
	2	0.07	0.04 (*p* = 0.025)	*F* (1.96) = 5.06 (*p* = 0.025)	—	0.11 [0.01, 0.21] (*p* = 0.025)
	3	0.10	0.07 (*p* = 0.002)	*F* (2.95) = 5.89 (*p* = 0.004)	0.15 [0.04, 0.26] (*p* = 0.002)	0.04 [−0.07, 0.15] (*p* = 0.55)

## Discussion

This study systematically examined the associations of upper- and lower-body muscle strength with working memory performance and its neural substrates in older adults by integrating behavioral performance and multi-regional neuroimaging metrics. The hierarchical regression analyses revealed a clear, consistent, and theoretically meaningful pattern: normalized grip strength is a dominant and unique physiological marker associated with working memory performance (especially processing speed) and associated prefrontal cortical activation. This core finding, together with the accompanying patterns such as functional prefrontal hierarchy and cognitive load moderation, calls for moving beyond the simplistic question of “whether” muscle strength is associated with cognition to explore the underlying physiological and cognitive mechanisms.

### The Superiority of Normalized Grip Strength as a “Hub Marker” Associated with Bodily-Brain Health

A key finding of this study is that normalized grip strength showed a consistent and independent association with working memory performance and prefrontal neural activation, whereas the association of the normalized 30-s sit-up became non-significant after accounting for grip strength. This suggests that among older adults, normalized grip strength may be a more representative physiological marker than lower-body strength for reflecting the reserve of overall neurocognitive resources. These findings align with previous studies, which have reported that grip strength in older adults is closely linked to cognitive function, particularly in working memory and processing speed (Cai et al., 2023; Firth et al., 2018). Previous studies have shown that a 5 kg increase in grip strength is associated with a larger white matter volume and a reduced risk of future cognitive impairment (Duchowny et al., 2022).

The present study found that grip strength was a more sensitive behavioral marker associated with working memory performance than the 30-s sit-up. This discrepancy may be due to several factors. First, as an isolated measure of maximal voluntary contraction, the grip strength test might reflect more purely the drive capacity of the central nervous system, thus establishing a more direct link with working memory, which also relies heavily on central control. In contrast, the 30-s sit-up represents a composite functional movement whose performance is affected not only by lower extremity muscle strength but also by muscular endurance, balance, coordination, cardiopulmonary endurance, and even willpower, possibly diluting its specific association with working memory. Second, given that our participants were older adults aged 75 years or above who are likely to have low physical activity levels, a “floor effect” for lower extremity strength measurements or insufficient interindividual variability could also explain the lack of significant behavioral correlations observed. Future studies should use more accurate isokinetic dynamometers to assess lower extremity muscle strength and replicate these findings in broader geriatric populations.

### Prefrontal Functional Hierarchy and Cognitive Load Modulation

The prefrontal functional hierarchy response pattern observed—strongest associations in the dorsolateral prefrontal cortex, moderate in the ventrolateral prefrontal cortex, and weakest in the frontopolar area—can be further explained by neurovascular coupling and hemodynamic regulation.

#### Efficiency gradient of neurovascular coupling

Substantial evidence suggests inherent differences in neurovascular coupling efficiency among PFC sub-regions. The DLPFC, as a core executive region for working memory, has higher microvascular density and more efficient astrocyte-mediated neurovascular unit coupling than the more anterior FPA (Iadecola, 2017). These structural differences directly affect the signal-to-noise ratio and task specificity of blood oxygen level-dependent signals or hemoglobin concentration changes measured by fNIRS (Huppert et al., 2009). When muscle strength is associated with systemic hemodynamic optimization (e.g., improved cardiac output and cerebrovascular reactivity), this association manifests most prominently in the DLPFC region, which depends heavily on efficient neurovascular coupling.

#### Task-load dependence of hemodynamic responses

Our finding that significant associations were almost exclusively observed in the 1-back and 2-back tasks is highly consistent with the non-linear characteristics of hemodynamic responses. Neural activity under a basic cognitive load (e.g., 0-back) induces only a limited increase in metabolic demand, which likely remains within the stable range of cerebral autoregulation and is therefore less affected by differences in peripheral physiological states (such as cardiovascular function associated with muscle strength) (Buxton et al., 2004). However, when cognitive demand increases (1-back, 2-back), neural activity intensifies, metabolic demands rise significantly, and stronger functional hyperemia and neurovascular coupling responses are required. At this point, individual differences in hemodynamic regulation capacity—which are correlated with muscle strength—become prominent, manifesting as significant associations between brain activation levels and behavioral performance.

#### Potential pathways between muscle strength and cerebrovascular health

Growing evidence suggests bidirectional associations between muscle strength and cerebrovascular health. In addition to the mechanical effects of muscle contraction that promote venous return and cerebral perfusion, myokines released by muscle (e.g., irisin and brain-derived neurotrophic factor) may act directly on the cerebrovascular system to enhance endothelial function, angiogenesis, and blood–brain barrier integrity. These effects are likely most pronounced in higher cognitive cortices such as prefrontal regions, which are more sensitive to fluctuations in blood flow and energy supply. The strong DLPFC associations we observed may partly reflect a specific demand for vasculogenic neurotrophic support in this region.

#### Spatiotemporal properties of the haemodynamic signal

It is important to note that hemoglobin concentration changes measured with fNIRS reflect localized hemodynamic responses, with different temporal properties (delay of −6–8 s) and spatial specificity (penetration depth of −1–3 cm) compared to BOLD-fMRI. The hierarchical pattern observed suggests that muscle strength may preferentially affect brain areas requiring rapid, robust hemodynamic responses (such as DLPFC), but has less effect on regions with lower hemodynamic regulatory demands or different temporal characteristics (such as FPA). This distinction further supports a “selective neurovascular optimization” hypothesis.

#### Cross-validation with aging studies

In older populations, declines in prefrontal hemodynamic responsivity (particularly DLPFC) are closely associated with executive function deficits. Interestingly, grip strength decline is often used as an early biomarker of aging and cognitive decline (Bohannon, 2019). Our findings capture this association pattern earlier in young to middle-aged samples, suggesting that the relationship between muscle strength and frontal lobe hemodynamic efficiency may persist throughout adulthood and become more pronounced with age. This offers a lifespan perspective on intervention windows for maintaining or enhancing prefrontal neurovascular health through improved muscular strength.

### Dialogue with existing theories and practical implications

The findings reinforce known neurophysiological pathways linking physical activity and cognition. They support the view that physical activity benefits the brain through improvements in cardiovascular and metabolic health, and identify normalized grip strength as a concrete, easily assessed variable for clinical and community settings. This provides a rationale for developing interventions aimed at maintaining or improving muscle strength, particularly in the upper body, among older adults. Such interventions may be valuable in the context of slowing age-related cognitive decline, particularly in executive functions. Our results are also partially consistent with the neural efficiency hypothesis. It should be noted, however, that greater activation could reflect either more efficient processing or compensatory recruitment in the face of increased task demands. The observed association with grip strength suggests that baseline physiological resources may modulate this neural response, though the exact nature (efficiency vs. compensation) requires further investigation.

Muscle strength is a key health indicator. Beyond its association with cognitive function, higher muscle strength levels can significantly reduce all-cause mortality and fall risk. Currently, muscle strength levels among older adults in China are concerning, particularly the marked decline in grip strength among the oldest-old, which demonstrates gender differences. Older males show a more pronounced reduction than females, a pattern possibly related to sex differences in rates of skeletal muscle atrophy. Data from Shanghai's National Physical Fitness Surveys reveal a consistent decline, emphasizing insufficient muscle strength as a “weak link” in residents' physical fitness. This decline is partly attributable to reduced participation in heavy physical activities and, more importantly, a lack of strength training. Currently, a small fraction of the population engages in activities that build muscle strength. This suggests a widespread shortfall in strength training.

Studies show a positive correlation between grip strength and moderate-to-vigorous physical activity (MVPA) in men. Using an isotemporal substitution model, replacing 30 min of sedentary behavior with 30 min of MVPA significantly increased grip strength in older adults (Wu et al., 2022). There is a significant dose-response relationship between physical activity, sedentary time, and physical health, with MVPA yielding substantial benefits. Thus, increasing physical activity is an effective way to improve grip strength, thereby potentially supporting working memory function. Older adults are advised to increase physical activity and reduce sedentary behavior to improve physical function and maintain health. However, current physical activity levels among older adults in China are suboptimal. The U.S. Physical Activity Guidelines recommend that older adults engage in at least 150 min of moderate-intensity exercise or 75 min of vigorous-intensity exercise per week, along with at least two sessions of strength training (Department of Health Human Services US, 2018). According to the 2015 WHO Global Health Survey, physical activity compliance rates decline with age. Research indicates that compliance rates among older adults in China range from 25.3% to 47.44%, with disparities linked to residency, gender, age, and education level (Sun et al., 2013). Compliance rates are higher among rural residents, males, younger seniors, and those with higher education. The duration of MVPA and average daily steps tend to decrease with age. Geographic region and marital status also influence compliance rates.

## Limitations and future directions

This study has several limitations. First, chronic conditions such as cardiovascular disease, diabetes mellitus, and hypertension could affect the outcome; these factors should be considered confounding variables in future studies. Second, we mainly used the 10–20 system to define the prefrontal ROIs; however, there is a significant interindividual anatomical variation that can result in mislocalization. Finally, the study population consisted of octogenarians with a mean age of over 80 years, recruited mainly from nursing homes, which restricts the generalizability of the results to community-dwelling or younger elderly populations.

Reverse Causality. This cross-sectional study cannot determine the direction of the observed associations. It is equally plausible that better-preserved cognitive function enables individuals to maintain higher levels of physical activity and muscle strength, or that both share common underlying causes (e.g., overall health, genetics). Future longitudinal and intervention studies are needed to clarify these temporal and causal relationships.

## Conclusion

Through an integrated analysis of behavior and multi-regional neural activity, this study identifies normalized grip strength as a central marker associated with working memory function and its prefrontal neural basis in older adults. The findings show that: (1) normalized grip strength is a more representative marker associated with cognitive outcomes than lower body strength, highlighting its role as an integrative physiological health indicator; (2) the association between muscle strength and prefrontal activation shows a clear functional hierarchy and is closely coupled with cognitive task complexity, suggesting its association with the neural response of specific higher cognitive processes. These findings advance the discussion on “muscle strength and brain health” from general association to mechanistic exploration, highlighting the potential value of monitoring upper body muscle strength in clinical and public health practice in the context of supporting cognitive health in aging.

## Data Availability

The original contributions presented in the study are included in the article/supplementary material, further inquiries can be directed to the corresponding author/s.
